# Regulating international clinical research: an ethical framework for policy-makers

**DOI:** 10.1136/bmjgh-2020-002287

**Published:** 2020-05-26

**Authors:** Bernardo Aguilera, David DeGrazia, Annette Rid

**Affiliations:** 1 Department of Bioethics, The Clinical Center, National Institutes of Health, Bethesda, Maryland, USA; 2 Department of Philosophy, George Washington University, Washington, DC, USA

**Keywords:** health policy, health services research, clinical trial

## Abstract

The global distribution of clinical trials is shifting to low-income and middle-income countries (LMICs), and adequate regulations are essential for protecting the rights and interests of research participants in these countries. However, policy-makers in LMICs can face an ethical trade-off: stringent regulatory protections for participants can lead researchers or sponsors to conduct their research elsewhere, potentially depriving the local population of the opportunity to benefit from international clinical research. In this paper, we propose a three-step ethical framework that helps policy-makers to navigate this trade-off. We use a recent set of regulatory protections in Chile to illustrate the practical value of our proposed framework, providing original ethical analysis and previously unpublished data from Chile obtained through freedom of information requests.

**Summary box**

- The global distribution of clinical trials is shifting to low-income and middle-income countries (LMICs), and adequate regulations are essential for protecting research participants.
- Policy-makers in LMICs often face an ethical trade-off: stringent regulatory protections can lead sponsors to conduct their research elsewhere, depriving LMICs of the potential benefits of international research.
- We develop a three-step ethical framework to guide policy-makers in dealing with key ethical considerations involved in navigating this trade-off.
- Using a recent set of regulatory protections in Chile, we illustrate the practical value of our proposed framework, providing original ethical analysis and previously unpublished data.
- The proposed ethical framework helps policy-makers strike an appropriate balance between protecting research participants and reaping the benefits of appropriate clinical research.

## Introduction

The global distribution of clinical trials is changing. Trials are shifting to low-income and middle-income countries (LMICs), where markets are expanding; participants are easily recruited; and research costs remain low.^1 2^ This shift has raised concerns about sponsors and researchers ‘outsourcing’ trials and possibly taking unfair advantage of vulnerable populations.^3^ For example, some researchers from high-income countries (HICs) have been criticised for using ‘double standards’ by conducting trials in LMICs that would not be ethically approvable in their home countries, such as placebo-controlled trials that withhold or delay the best proven treatments.^4 5^

In response, some LMICs have introduced stringent regulatory protections for clinical trial participants. For example, in 2012, India required that sponsors provide free clinical care to participants injured during a trial, whether or not the injury was research-related.^6^ However, stringent protections can lead sponsors to conduct their research elsewhere, depriving LMICs of the potential benefits of hosting studies. In India, for instance, clinical trial registrations fell sharply after the new protections were introduced.^6^

At the time, the dominant reaction was negative: India might lose the benefits of international research.^7^ Indeed, international research is often regarded as an important means of improving infrastructure, training and enhancing economic activity in LMICs.^8 9^ Thus, the ethical challenge is to balance the goals of protecting research participants and reaping the benefits of appropriate clinical research.^10^ This paper explores Chile’s recent experience to address this issue and develops ethical guidance for policy-makers. While especially useful for policy-makers in LMICs, our proposed ethical framework can also guide policy-makers in HICs who seek to protect participants while promoting beneficial research.

## Emerging economies and research regulation: the case of Chile

Clinical trials have expanded most rapidly in ‘emerging economies’, where appropriate healthcare infrastructure and skilled research workforces attract private sponsors.^11–13^ As an emerging economy with a high density of externally sponsored clinical trials,^14^ Chile helpfully illustrates how countries in the current landscape of international research face ethical trade-offs in clinical trial regulation.

Chile passed its first law on clinical research in 2006. Criticised for insufficiently focusing on ‘the protection of persons’,^15^ this law has been succeeded by further research regulations. In 2015, Law 20.850 introduced changes regarding compensation for research-related injuries and post-trial access to treatments that are remarkably stringent.^16^ As in other Latin American countries, the regulations are more stringent than international guidelines (table 1), indicating a regional trend towards greater protections for research participants.^17^

**Table 1:** Comparison of the WMA’s Declaration of Helsinki,^59^ the CIOMS‘s International Ethical Guidelines for Health-related Research Involving Humans^21^ and Chile’s Law 20.850^16^

WMA, Declaration of Helsinki (2013)	CIOMS, International Ethical Guidelines for Health-related Research Involving Humans (2016)	Chile’s Law 20.850 (2015)
*Compensation for research-related injuries*		
Article 15 ‘Appropriate compensation and treatment for subjects who are harmed as a result of participating in research must be ensured’.	Guideline 14 ‘Sponsors and researchers must ensure that research participants who suffer physical, psychological or social harm as a result of participating in health-related research receive free treatment and rehabilitation for such harms, as well as compensation for lost wages, as appropriate. Such treatment and compensation are owed to research participants who are harmed physically, psychologically or socially, as a consequence of interventions performed solely to accomplish the purposes of research, regardless of fault’.	Article 111 E The sponsor ‘will be liable for injuries caused by the study, even when they result from facts or circumstances that could not have been foreseen or avoided according to the technical and scientific knowledge available at the time the injuries were produced. Moreover, once the injury is confirmed, it will be presumed that it was produced by the study. The opportunity to prosecute the responsible will expire ten years after the injury manifests’.
		Article 111 F Sponsors ‘will be obliged to have civil liability insurance, in accordance with the regulations dictated by the Ministry of Health…’.
*Post-trial access to treatments*		
Article 34 ‘In advance of a clinical trial, sponsors, researchers and host country governments should make provisions for post-trial access for all participants who still need an intervention identified as beneficial in the trial’.	Guideline 6 ‘When participants’ health needs during and after research cannot be met by the local health infrastructure or the participant’s pre-existing health insurance, the researcher and sponsor must make prior arrangements for adequate care for participants with local health authorities, members of the communities from which persons are drawn, or nongovernmental organizations such as health advocacy groups … When access is provided after the research to investigational interventions that have demonstrated significant benefit, the provision may end as soon as the study intervention is made available through the local public health-care system or after a predetermined period of time that the sponsors, researchers and community members have agreed before the start of a trial’.	Article 111 C ‘Clinical trial subjects will have the right, once the trial is over, that the [sponsor] … continues to provide the [investigational or, at a later, point, the licensed] treatment free of charge and for as long as its therapeutic utility remains, according to the respective research protocol’.

Excerpts from Law 20.850 have been translated by BA. To date, there are no procedural rulings on Law 20.850 that could provide further context for its interpretation.

CIOMS, Council for International Organizations of Medical Sciences; WMA, World Medical Association.

## Stricter protections, fewer trials?

**Figure 1:**
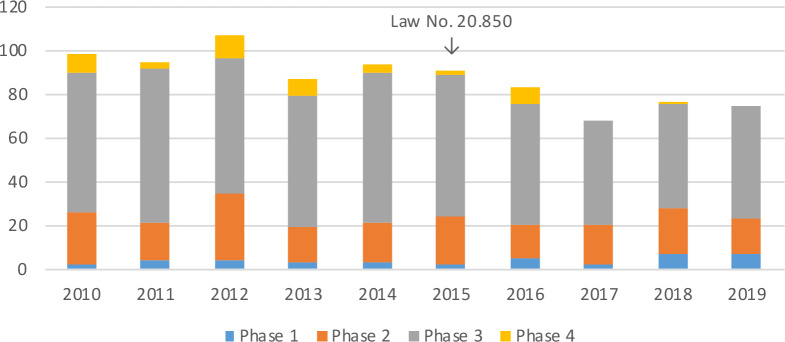
Number and phase of clinical trials registered in Chile between 2010 and 2019. Research sponsors are legally required to register all trials of drugs that have not been licensed by the Chilean Institute of Public Health (equivalent to the Food and Drug Administration in the USA) or are used outside their licensed indication. The arrow indicates when Chile’s Law 20.850 was enacted. The total number of trials (2010–2019) was N=876. Three trials (n=2 (2011), n=1 (2017)) were excluded since they were not reported as corresponding to phases 1–4. Data were obtained by freedom of information requests.

Several Chilean parliament members and academics raised concerns that Law 20.850 might disincentivise clinical research.^18^ Data from the Chilean Institute of Public Health, obtained through freedom of information requests, seem to support these concerns. Based on the Institute’s mandatory registry of clinical trials involving investigational drugs, we found that the mean number of trials registered per year dropped from 95.5 (SD 6.9) before Law 20.850 was enacted to 75.8 (SD 6.2) (figure 1). The decline occurred mainly in phase 3/4 clinical trials, which decreased from an average of 71.2 (SD 2.9) between 2010 and 2015 to 53 (SD 6.9) between 2016 and 2019. Clinical trial sponsorship remained stable during both time periods, with a mean of 97% trials per year sponsored by pharmaceutical companies from outside Chile.

Can the new regulatory protections for participants explain this decline in international clinical research? This is a difficult question, as the absolute number of trials in Chile is low and our observation period is relatively short. Nonetheless, several considerations support a causal relationship.

The decrease in clinical trial numbers immediately followed the introduction of Law 20.850, with new trial registrations remaining below 87 annually, the lowest number of registrations observed in any of the previous years (figure 1). The sharpest decline has occurred in phase 3/4 clinical trials, for which the new regulatory protections are most relevant. Phase 3/4 trials can involve large numbers of participants, increasing the likelihood that sponsors must compensate for injuries. Study drugs are also more likely to prove beneficial in later trial phases, triggering post-trial access requirements. The increased prospect of substantial additional costs might have led sponsors to discontinue trials or move them to countries with less stringent regulatory protections, as occurred in India.^10^ Finally, other factors do not seem to explain the decline in clinical trial registrations in Chile. There was neither a wider downward trend in clinical research activity (indeed, global trial numbers have grown steadily in recent years^19^) nor wider economic or political instability in Chile that could have deterred research sponsors, and the Chilean clinical trial market is not yet considered saturated.^20^

## Ethical trade-offs between protecting participants and reaping research benefits

Most laws and regulations have some negative consequences. In the present case, Law 20.850 might have disincentivised international research sponsors from registering new clinical trials in Chile. We recommend that policy-makers balance the goals of protecting research participants and reaping the benefits of appropriate clinical research using the following three-step framework (table 2). Using Law 20.850 as an example (table 1), we discuss how policy-makers should evaluate the need to revise existing protections.

**Table 2:** A three-step ethical framework for evaluating existing regulatory protections for clinical research

Guiding questions	Concrete tasks	
1. Do the regulatory protections have a sound ethical rationale?	Determine whether the regulatory protections are prima facie ethically defensible.	
	Specifically, consider whether the protections are consistent with widely accepted ethical standards for clinical research; whether they protect participants’ rights and interests; and whether the protections meet other ethical criteria, such as a just and fair distribution of research benefits and burdens.	
	A negative answer to all these questions provides a strong indication that the protections should be revised or revoked. However, the next steps of the framework should still be followed in order to confirm this answer and decide whether and how the protections should be revised, all things considered.	
2. What are the benefits and costs of implementing the regulatory protections?	Survey all the relevant facts regarding the effects of implementing the regulatory protections.	
	Specifically, examine systematically who benefits from the protections and who incurs costs, including how significant the benefits and costs are. Have the affected people fared better or worse after the protections were introduced, as compared with how they would have fared if the protections had not been introduced?	
	Be sure to consider the benefits and costs for everyone affected, based on the list provided below. (Note that listed potential benefits can turn into potential costs when a given regulatory protection leads to a decline in clinical research activity and, consequently, to forgone benefits; conversely, listed potential costs can turn into potential benefits.)	
	Research participants	Potential clinical benefits from the research intervention (during and after the trial if post-trial access to proven beneficial interventions is provided)
		Potential clinical benefits from improved clinical care as part of the research (‘inclusion benefits’)
		Potential clinical benefits from ancillary care (eg, following up on diagnoses based on research tests, treating conditions unrelated to the study’s aims)
		Potential clinical costs or harms (eg, research-related injuries)
		Potential psychological benefits (eg, feelings of altruism)
		Potential psychological costs (eg, anxiety from undergoing research procedures or receiving research results)
		Potential social benefits (eg, social recognition)
		Potential social costs (eg, stigma or discrimination)
		Potential financial benefits from monetary compensation
		Potential financial costs (eg, transportation costs, lost wages, treatment costs for research-related injuries)
	Patients	Potential clinical benefits from access to new interventions
		Potential clinical benefits from research-related improvements in the quality of routine clinical care
		Potential clinical benefits from advances in scientific or medical knowledge that address local health needs or priorities (primarily fostered through local research capacity building)
		Potential clinical costs or harms if qualified clinicians are diverted from routine clinical care to clinical research
	Wider community	Potential financial benefits from cost savings for healthcare payers (if research sponsors cover study treatments)
		Potential financial benefits from research-related economic activity (eg, research-related jobs or bonuses) and tax revenues
3. Are the regulatory protections justified, all things considered?	Consider whether the regulatory protections are, all things considered, ethically justified.	
	Specifically, weigh the benefits against the costs of the regulatory protections, consider to what extent the distribution of benefits and costs across different population groups promotes or curtails justice, and judge whether the costs to individuals or certain groups amount to a violation of their rights.	
	If the benefits of the regulatory protections do not outweigh the costs, the protections create new injustices or exacerbate existing ones, or the protections violate the rights of individuals or certain groups, there is reason to revise or amend the protections.	
	If none of these ethical problems is evident and the protections’ benefits seem to outweigh the costs, then the regulatory protections are ethically justified.	

Online supplementary appendix 1 provides a summary of how the framework can be applied to the Chilean Law 20.850. Online supplementary appendix 2 provides a slightly modified version of the framework for evaluating new regulatory protections under consideration (rather than existing regulatory protections).

10.1136/bmjgh-2020-002287.supp1 Supplementary data

10.1136/bmjgh-2020-002287.supp2 Supplementary data

### Step 1: do the regulatory protections have a sound ethical rationale?

Policy-makers should determine, first, whether the regulatory protections are prima facie ethically defensible. Are the protections consistent with widely accepted ethical standards for clinical research? Do they protect participants’ rights and interests, such as the right to be free of non-consensual experimentation or the interest in maintaining good health? Do the protections meet other ethical criteria, such as a just and fair distribution of research benefits and burdens?

A negative answer to all these questions provides a strong indication that the protections should be revised or revoked. For example, if a regulation excluded illiterate populations from research because of concerns about their decisional capacity, it should be revised. Because literacy is not necessary for making voluntary and informed decisions, excluding these populations prevents them from exercising their rights and, in some trials, promoting their interests by enrolling in potentially beneficial research. A blanket exclusion would also undermine principles of fair participant selection and conflict with recognised international guidelines.^21^ However, even in prima facie clear-cut cases, policy-makers should engage in an in-depth ethical analysis using our proposed framework. Such analysis can help to decide whether and how a given regulation should be revised, all things considered.

In the present case, the provision of compensation for research-related injuries and post-trial access to proven beneficial treatments are generally consistent with sound ethical standards. However, a careful analysis of Law 20.850 suggests that reasonable people would likely disagree about how the law should spell out these standards.

Law 20.850 requires research sponsors to obtain insurance that compensates for research-related injuries, ‘even when they result from facts or circumstances that could not have been foreseen or avoided according to the technical and scientific knowledge available at the time’.^16^ This ‘no-fault’ compensation scheme is widely considered ethically sound because it protects participants by reducing the harms of research-related injuries and helps to distribute research risks and potential benefits fairly.^22^ However, Law 20.850 goes beyond ‘no-fault’ compensation by stipulating that ‘once the injury is confirmed, it will be presumed that it was produced by the study’.^16^ The law only specifies that ‘the opportunity to prosecute … will expire ten years after the injury manifests’, leaving open the possibility of prosecuting injuries that manifest at any point after a study.^16^ These provisions arguably place an excessive burden on research sponsors, as they might be held liable for conditions that arise unrelated to the research.

Law 20.850 also mandates that sponsors provide participants the investigational treatment—or, at a later point, the licensed drug—‘free of charge and for as long as its therapeutic utility remains’ after the study has ended.^16^ These provisions are prima facie sound insofar as they protect the interests of patients and compensate for their participation in research. However, reasonable people might disagree as to whether the *sole* responsibility for providing post-trial access to treatments should be placed on sponsors. For example, when local authorities or institutions can contribute to ensuring post-trial access, the resulting burden on sponsors could reasonably be considered excessive. Moreover, it can be argued that the supply of effective treatments is sustainable only when sponsors and local actors actively engage in a collaborative partnership.^23 24^

In sum, while a prima facie case for Law 20.850 can be made, certain aspects of the protections it sets out can reasonably be considered to place excessive burdens on researchers and sponsors.

### Step 2: what are the benefits and costs of implementing the regulatory protections?

After scrutinising the prima facie ethical case for the given regulatory protections, policy-makers should survey all the relevant facts regarding their effects. Specifically, policy-makers should examine systematically who benefits from the protections and who incurs costs, including how significant the benefits and costs are. We understand benefits and costs broadly: have the affected people fared better or worse after the protections were introduced, as compared with how they would have fared if the protections had not been enacted? This analysis should encompass everyone affected: not only research participants but also patients and the wider community who can receive benefits or incur costs from international clinical research (table 2).

We limit our discussion of Law 20.850 to the Chilean population, as this is the primary focus for Chilean policy-makers and the law’s global impact is less certain. However, depending on policy-makers’ needs, our framework can be applied on a national, regional, or global level.

#### Research participants

Although participants are the intended beneficiaries of Law 20.850, we are unaware of empirical data demonstrating that more participants have received appropriate compensation for research-related injuries or post-trial access to beneficial interventions. However, even assuming good-faith implementation of the law, it is unlikely that it made participants substantially better off than they otherwise would have been. Previous regulations already had a ‘no fault’ scheme of compensation for research-related injuries (though less stringent),^25^ and post-trial access to investigational drugs was authorised (though not required).^26^ Since no more than two-thirds of investigational drugs prove beneficial even in late-phase trials,^27^ this suggests that the law’s benefits to research participants are limited.

*Potential* research participants might be worse off if the decline in clinical trials following Law 20.850 deprived them from the benefits of enrolment. Yet evidence suggests that clinical outcomes are neither better nor worse in clinical trials than in routine care.^28^ Moreover, the Chilean healthcare system provides meaningful access to health services,^29^ so that potential participants’ clinical needs can reasonably be met outside the research context. And the financial compensation that potential participants might obtain by enrolling in research is typically small and limited to reimbursements for time or expenses incurred—particularly in phase 3/4 trials,^30^ which declined most sharply after Law 20.850 was introduced. Thus, it seems unlikely that the law made potential participants noticeably worse-off.

Conversely, potential participants might be better off if the decline in trials meant they incurred fewer research-related risks. While robust evidence on the psychological and social risks of participating in research is lacking, the risk of serious health-related harm is generally low in late-phase clinical trials.^31^ Taken together, this suggests that Law 20.850 has led to limited benefits for clinical trial participants. Additionally, the law has likely had no noticeable impact on potential participants who have been deprived of the opportunity to enrol in research.

#### Patients

Due to the decline in clinical trials following Law 20.850, patients might be worse off due to a reduced access to successful products. However, these costs to patients are likely limited. New drugs can be licensed in Chile whether or not they have undergone local testing.^32^ Moreover, even if local data were required for licensure, the research currently being conducted does not address patients’ most pressing health needs. According to our data, at most 13.5% of the trials registered in Chile between 2016 and 2019 addressed one of the top 10 causes of disability-adjusted life years in the country^33^ (table 3). Moreover, around 30% of investigational drugs that undergo pivotal trials in Latin America are not licensed in the countries where they are tested, and licensed products remain unaffordable for most of the local population.^34^

**Table 3:** Top 10 causes of death and disability combined in Chile,^33^ matched with the number and percentage of clinical trials that address the given cause between 2016 and 2019 (ie, after Law 20.850 was introduced)

Top 10 causes of death and disability combined	Number of clinical trials addressing the given cause
(Chile, 2017)	(Chile, 2016–2019; n (% of 303 registered trials))
1. Low back pain	9 (3)
2. Ischaemic heart disease	9 (3)
3. Stroke	3 (1)
4. Diabetes	7 (2.3)
5. Cirrhosis	3 (1)
6. Neonatal disorders	9 (3)
7. Depressive disorders	1 (0.3)
8. Headache disorders	0
9. Road injuries	0
10. Anxiety disorders	0
Total	41 (13.5)

We used a broad interpretation of when trials address a given cause of death and disability, meaning the trial could address the given cause either directly or indirectly.

Further, with a decline in clinical trial numbers, fewer patients might benefit from the improvements in clinical care that result from the organisational culture, systems and expertise fostered by research.^35^ However, in Chile, the associated costs are likely limited. Evidence about research-related improvements in clinical care comes mostly from HICs, which may be better equipped to implement quality improvements.^36^ Moreover, many reports of research-related clinical capacity building involve publicly funded trials.^37^ It is unclear to what extent private sponsors—which fund 97% of clinical trials in Chile—achieve similar results. Likewise, the decline in clinical trial numbers likely had no noticeable benefits for patients from research staff moving to provide routine clinical care, as Chile has no shortage of trained clinicans.^38^

Finally, patients might be worse off due to the decline in clinical trials and the resulting loss in research capacity building for the development of locally relevant drugs. Yet, only 2.5% of publications from externally sponsored trials in Chile list a local researcher as a coauthor, suggesting that local research involvement is limited.^39^ Moreover, according to our data, more than 70% of clinical trials in Chile are phase 3/4 trials. Because these trials test existing hypotheses, rather than generating new ones, they provide local researchers limited opportunities for developing their research skills.^40^ Indeed, Chilean researchers worry that local ‘efforts for building a scientific and entrepreneurial development in Chile would … be wiped out’ because the requirements of Law 20.850 are too difficult for local researchers and sponsors to meet.^41^

In sum, the decline in clinical trials after Law 20.850 was introduced does not seem to have led to major costs or harms for patients.

#### Wider population

The wider population might be worse off economically if Law 20.850 deprived them of clinical trial activity and the associated financial and economic benefits. Privately sponsored trials can provide drugs and services free of charge and thereby save costs for healthcare payers.^42 43^ While there is no corresponding evidence from Chile, we expect some such cost savings to occur. According to some reports from HICs, clinical research activities also significantly boost the national economy.^44 45^ In Chile, pharmaceutical industry sources claim that private investment is ‘annually around US$30 million in clinical studies and provides jobs to more than 2100 Chilean professionals and technicians’.^46^ Moreover, local healthcare professionals might receive compensation from pharmaceutical companies for recruiting participants.^47^ Law 20.850 therefore likely led to noticeable economic costs for the wider population. However, considering a labour force of >9 million people and a GDP of >$277 billion,^48^ these costs seem relatively small on a national scale.

### Step 3: are the regulatory protections ethically justified, all things considered?

In the final step of our proposed framework, policy-makers should consider whether the given regulatory protections are, all things considered, ethically justified. While step 2 mostly involves descriptive fact-finding, step 3 involves ethical judgement in light of the relevant facts. Specifically, policy-makers should weigh the benefits of the given protections against the costs. They should then consider how these benefits and costs are distributed across different population groups, and to what extent that distribution creates new injustices or exacerbates existing ones—for example, by worsening the lot of the least advantaged in society.^49^ Finally, policy-makers should judge whether the costs to individuals or certain groups amount to a violation of their rights or vital moral safeguards, such as the right not to be exposed to risks and burdens that are disproportionate to the potential benefits of the research.^50–55^

If the benefits do not outweigh the costs, the protections create new injustices or exacerbate existing ones, or the protections violate the rights of individuals or certain groups, there is reason to revise or amend the protections. If, however, none of these ethical problems is evident and the protections’ benefits seem to outweigh the costs, then the protections are ethically justified. Table 4 provides examples of rights and justice considerations that are relevant for making these judgements. However, because rights and justice considerations are highly context-specific, policy-makers should carefully (and critically) consider the given legal, political and social contexts.

**Table 4:** Examples of rights and justice considerations when evaluating regulatory protections for clinical research

Rights considerations	Application in the context of clinical research
Right to have one’s interests and well-being prevail over the sole interest of society or science	Right to have the risks and burdens of research participation minimised and not to be deprived of medically necessary procedures
	Right not to be exposed to risk and burdens that are disproportionate to the potential benefits of the research; if the research does not have the potential to benefit one’s health, it must entail no more than acceptable risk and acceptable burden
	Right to be enrolled in research only when it is carried out under the supervision of a clinical professional who possesses the necessary qualifications and experience
	*For persons not able to provide consent*: right to be enrolled in research only when it has the potential to produce real and direct health benefits, or when it entails minimal risk or burden, and the research has the aim of benefiting other persons in the same age or disease category
Right to be free from non-consensual experimentation	Right to be enrolled in research only with one’s explicit free and informed consent to research
	Right to withdraw from research freely at any time and for any reason without disadvantage or prejudice
	*For persons not able to provide consent*: right to be enrolled in research only with the authorisation of a legal representative, to have one’s previously expressed wishes relating to research taken into account, and to be involved to the greatest extent possible in the decision-making process; research should be undertaken only if the person concerned does not object
Right to non-discrimination and non-stigmatisation	Right to have one’s integrity, other rights and fundamental freedoms with regard to research respected without discrimination or stigmatisation on any grounds (eg, without distinction of race, religion, political belief, sexual orientation, economic or social condition)
Right to have any personal information collected treated confidentially	Right to have any personal information collected during research considered as confidential and treated according to the rules relating to the protection of privacy
Right to know about any information collected	Right to know about any information collected about one’s health
	Right to access other personal information collected
	Right to have one’s wish not to be informed respected
Right to receive research results	Right to receive the conclusions of the research on request
	Right to receive information relevant to one’s current or future health or quality of life, within a framework of healthcare or counselling
Right to receive compensation for harm	Right to fair compensation for harm suffered as a result of participation in research
Right to independent ethics review	Right to be enrolled in research only after independent examination by an ethics committee has confirmed that the dignity, rights, safety and well-being of research participants are protected
Right to enjoy the benefits of scientific progress	Right to access the benefits of science and its applications, including scientific knowledge
	Right to have opportunities to contribute to the scientific enterprise and the freedom indispensable for scientific research (eg, freedom from political and other interference)
	Right to participate in science-related decision-making
**Justice considerations**	**Application in the context of clinical research**
Equal moral standing	The dignity and rights of all research participants are to be respected so that all participants are treated justly and fairly
Priority to the worst off*	Special weight should be given to enhancing research benefits, and reducing research costs, to those who are least advantaged

The examples are derived from key international documents regarding human rights and health^50–55^ and relevant literature on health justice.^49^ Note that the referenced documents do not include a right to post-trial access, although this could potentially be derived from other rights listed in the table (eg, the right to receive research results and the right to enjoy the benefits of scientific progress).

*A ‘moderate prioritarian’ view—which gives substantial, but not absolute priority to the worst off—is widely endorsed because it can be justified drawing on egalitarian, prioritarian, sufficientarian and utilitarian theories of distributive justice.^49^

All things considered, our analysis suggests that the decline in clinical trials after Law 20.850 was introduced likely resulted in limited benefits to research participants and limited costs to patients, but noticeable costs to the wider population (online supplementary appendix 1). These findings support the common idea that LMICs can be made worse off by regulatory protections that disincentivise international clinical research, but with four important qualifications. First, since we did not conduct a formal cost–benefit analysis, our findings remain tentative. Second, both benefits and costs appear to be relatively small and might not be significant overall. This could be different in other countries, especially those with higher trial numbers. Third, in an emerging economy like Chile, costs resulting from fewer clinical trials seem to be mainly due to economic losses, instead of forgone benefits to participants and patients. Again, this could be different in other contexts, for example, resource-poorer LMICs where trials might help to meet basic health needs. Finally, both the benefits and costs of Law 20.850 could change over time. For example, clinical trial numbers might recover as sponsors adapt to the law, raising the question whether the limited aggregate costs during an expected transition period are a ‘price worth paying’ for the additional protections for participants.

Our findings also highlight the important fact that the benefits and costs of research regulations can be of multiple kinds and degrees, and that they are distributed across different populations. This brings out considerations of rights and justice. Partly because the benefits and costs of Law 20.850 are relatively small, our analysis does not suggest that it led to injustices or rights violations. However, where protections have more important benefits and costs, policy-makers should evaluate their distribution and moral significance by drawing on relevant notions of justice and rights.

Finally, our analysis raises concerns about how international clinical research in Chile is currently being conducted. Specifically, the costs of implementing Law 20.850 are limited mainly because the decline in clinical trials does not entail significant losses in research capacity building and research that is responsive to local health needs. This is cause for concern, given the potential of local research capacity to improve health and reduce health disparities in Chile.^56^

In sum, we believe that building a strong ethical case for revising Law 20.850 remains elusive. However, dispensing with those aspects of the law that can be reasonably considered too burdensome, while upholding appropriate protections regarding research-related injuries and post-trial access, might help to remove disincentives for conducting international clinical research in Chile. Any regulatory changes in this direction should ideally be associated with measures to actively promote local research capacity building and the conduct of research that is responsive to local health needs. For this to happen, Chilean policy-makers might strengthen research institutions, set clear research priorities, provide adequate research funding, and promote innovative partnerships with research sponsors.^57^

## Conclusion

Policy-makers should be aware of the ethical trade-offs involved in regulating clinical research. We have proposed a three-step ethical framework for balancing the goals of protecting research participants and reaping the benefits of hosting clinical trials. This framework guides policy-makers in integrating key ethical and practical considerations, including which population groups are affected by a given set of regulatory protections, how different types of benefits and costs are distributed across these groups, and whether the rights of individuals or groups are being violated. Our framework could helpfully be expanded, for example, by further specifying notions of justice and rights, or incorporating elements of more formal cost–benefit analysis.^58^ However, policy-makers often have to make decisions with limited information and under time constraints. In this context, our framework provides useful ethical insights, which policy-makers might need to weigh with political or other considerations. Of note, although we use Chile as an illustrative example, our framework applies in LMICs as well as HICs and can be used to evaluate the need to revise existing protections with a national, regional or global scope. Similarly, the framework can be adapted so that policy-makers can evaluate new protections under consideration (online supplementary appendix 2).
