# Supplementary material for: Regulating international clinical research: an ethical framework for policy-makers
Source: BMJ Glob Health. 2020 May 26;5(5):e002287. doi: 10.1136/bmjgh-2020-002287 (PMC7259867; doi:10.1136/bmjgh-2020-002287)
Supplement: Supplementary data [file bmjgh-2020-002287supp001.pdf]

Appendix 1: The three-step framework for evaluating regulatory protections applied to the provision of compensation for research-related injuries and post-trial access as specified in Chile's 2015 Law No. 20.850.

Guiding question	Concrete tasks
1. Do the regulatory protections have a sound ethical rationale?	Both regulatory protections are generally consistent with sound ethical standards and aimed at protecting participants' rights and interests.<br><br>Overall, there is no indication that the protections should be revised or revoked. However, certain aspects of the protections might reasonably be considered to place excessive burdens on researchers and sponsors.
2. What are the benefits and costs of implementing the regulatory protections?	Research participants have likely experienced limited clinical and financial benefits from the protections. Potential participants have likely experienced no noticeable clinical and financial benefits, and incurred no noticeable clinical and financial costs, from the protections.<br><br>Patients have likely incurred limited clinical costs or harms from the protections because of the resulting decline in clinical research activity, and hence the resulting decline in access to new interventions, research-related improvements in the quality of routine clinical care, and local research capacity-building for addressing local health needs or priorities.<br><br>The wider community has likely incurred noticeable economic costs from the protections because of the resulting decline in clinical research activity.<br><br>*Note that this analysis focuses on the Chilean population only.*
3. Are the regulatory protections justified, all things considered?	All things considered, the regulatory protections have likely resulted in limited benefits to research participants and limited costs to patients, but noticeable costs to the wider population. This suggests that the costs of the protections outweigh its benefits.<br><br>However, the benefits and costs of implementing the protections appear to be relatively small and might not be significant overall. Importantly, the costs seem limited mainly because the decline in clinical research activity has not led to significant losses in research capacity building and research that is responsive to local health needs. Implementing the protections does not seem to have led to injustices or rights violations.<br><br>All things considered, there is no strong ethical case for revising the protections. However, the ethical justification for the protections may be enhanced by dispensing with those aspects that can reasonably be considered too burdensome, while upholding appropriate safeguards for participants' rights and interests. Any regulatory changes in this directions should ideally be associated with measures to actively promote local research capacity building.
