# Supplementary material for: Regulating international clinical research: an ethical framework for policy-makers
Source: BMJ Glob Health. 2020 May 26;5(5):e002287. doi: 10.1136/bmjgh-2020-002287 (PMC7259867; doi:10.1136/bmjgh-2020-002287)
Supplement: Supplementary data [file bmjgh-2020-002287supp002.pdf]

Appendix 2: A three-step framework for evaluating regulatory protections for clinical research that are under consideration (ie, not yet implemented).

**Guiding question**	**Concrete tasks**	
1. Do the regulatory protections have a sound ethical rationale?	Determine whether the regulatory protections are *prima facie* ethically defensible.<br><br>Specifically, consider whether the protections are consistent with widely accepted ethical standards for clinical research; whether they protect participants’ rights and interests; and whether the protections meet other ethical criteria, such as a just and fair distribution of research benefits and burdens.<br><br>A negative answer to all these questions provides a strong indication that the protections should be revised or revoked. Otherwise, the next steps of the framework should be followed.	
2. What are the benefits and costs of implementing the regulatory protections?	Foresee all the relevant potential effects of implementing the regulatory protections.<br><br>Specifically, estimate systematically who might benefit from the protections and who might incur costs, including how significant the estimated benefits and costs are. Would the affected people fare better or worse if the protections were introduced, as compared to how they would fare if the protections were not introduced?<br><br>Be sure to consider the potential benefits and costs for everyone affected, based on the list provided below. (Note that listed potential benefits can turn into potential costs when a given regulatory protection leads to a decline in clinical research activity and, consequently, to forgone benefits; conversely, listed potential costs can turn into potential benefits.)	
	Research participants	Potential clinical benefits from the research intervention (during and after the trial if post-trial access to proven beneficial interventions is provided)
		Potential clinical benefits from improved clinical care as part of the research (“inclusion benefits”)
		Potential clinical benefits from ancillary care (eg, following up on diagnoses made based on research tests, treating conditions that are unrelated to the study's aims)
		Potential clinical costs or harms (eg, research-related injuries)
		Potential psychological benefits (eg, feelings of altruism)
		Potential psychological costs (eg, anxiety from undergoing research procedures or receiving research results)
		Potential social benefits (eg, social recognition)
		Potential social costs (eg, stigma or discrimination)
		Potential financial benefits from monetary compensation
		Potential financial costs (eg, transportation costs, lost wages, treatment costs for research-related injuries)
	Patients	Potential clinical benefits from access to new interventions
		Potential clinical benefits from research-related improvements in the quality of routine clinical care

		Potential clinical benefits in the longer term from advances in scientific or medical knowledge that address local health needs or priorities (primarily fostered through local research capacity building)
		Potential clinical costs or harms if qualified clinicians are diverted from routine clinical care to clinical research
	Wider community	Potential financial benefits from cost-savings for healthcare payers (if research sponsors cover study treatments)
		Potential financial benefits from research-related economic activity (eg, research-related jobs or bonuses) and tax revenues
3. Are the regulatory protections justified, all things considered?	Consider whether the regulatory protections are, all things considered, ethically justified.<br><br>Specifically, weigh the estimated benefits against the estimated costs of the regulatory protections, consider to what extent the distribution of benefits and costs across different population groups might promote or curtail justice, and judge whether the costs to certain individuals or groups might amount to a violation of their rights.<br><br>If, in this hypothetical scenario, the benefits of the regulatory protections do not outweigh the costs, the protections create new injustices or exacerbate existing ones, or the protections violate the rights of certain individuals or groups, there is reason to revise or amend the protections.<br><br>If none of these ethical problems is evident and the protections' estimated benefits seem to outweigh the estimated costs, then implementing the regulatory protections is ethically justified.	
